# A Microtester for Measuring the Reliability of Microdevices in Controlled Environmental Conditions

**DOI:** 10.3390/mi12050585

**Published:** 2021-05-20

**Authors:** Yunjia Li, Weitao Dou, Chenyuan Zhou, Xinyi Wang, Aijun Yang, Yong Zhang, Dayong Qiao

**Affiliations:** 1 School of Electrical Engineering, Xi’an Jiaotong University, Xi’an 710049, China; dou_wt@stu.xjtu.edu.cn (W.D.); zhouchenyuan@stu.xjtu.edu.cn (C.Z.); xywang@stu.xjtu.edu.cn (X.W.); yangaijun@xjtu.edu.cn (A.Y.); zhyong@mail.xjtu.edu.cn (Y.Z.); 2 Key Laboratory of Micro/Nano Systems for Aerospace, Ministry of Education, Northwestern Polytechnical University, Xi’an 710072, China

**Keywords:** temperature and humidity control, decoupling control, reliability test, microtester

## Abstract

A miniaturized reliability test system for microdevices with controlled environmental parameters is presented. The system is capable of measuring key electrical parameters of the microdevices while controlling the environmental conditions around the microdevices. The test system is compact and thus can be integrated with standard test equipment for microdevices. By using a feed-forward decoupling algorithm, the presented test system is capable of generating a temperature range of 0–120 °C and a humidity range of 20–90% RH (0–55 °C), within a small footprint and weight. The accuracy for temperature and humidity control is ±0.1 °C and ±1% RH (30 °C), respectively. The functionality of the proposed test system is verified by integrating it with a piezo shaker to test the environmental reliability of an electromagnetic vibration energy harvester. The proposed system can be used as a proof-of-technology platform for characterizing the performance of microdevices with controlled environmental parameters.

## 1. Introduction

Reliability of microelectromechanical system (MEMS) is one of the essential factors that determines the development time and time-to-market of the devices. Besides the design and the fabrication processes, environmental factors such as temperature, humidity and vibration are important aspects that influence the in-use reliability of MEMS devices [1]. In addition, environmental factors are known acceleration factors for accelerated reliability test of MEMS. For instance, temperature is often used to accelerate creep, electrical short/open-circuit, charging and corrosion failures, humidity is commonly used to accelerate cyclic fatigue, stiction and charging failures, and vibration is usually used to accelerate stiction and shock/vibration failures [1].

A great deal of work has been carried out to characterize MEMS reliability under different environmental conditions. For instance, Pustan et al. investigated the influence of temperature and humidity on the micromirror devices by using the combination of an Atomic Force Microscope and an environmental chamber [2]. It was discovered that the stiffness of the micromirror is reduced by 46% in the temperature range of 20 °C to 140 °C. Zhang et al. used the standalone environmental chamber to study the influence of temperature and humidity on the linearity and sensitivity of RF MEMS power sensors [3]. The test temperature and humidity ranges are 5 °C to 75 °C and 25% to 95% RH, respectively. Jan et al. proposed a test platform that combines a double-layer environmental chamber and a vibration shaker to study the effect of temperature and humidity on the resonant frequency of a CMOS-MEMS paddle resonator [4]. Resonant frequency drops of 6.9 Hz and 1.3 Hz were observed in the temperature range of 25–80 °C and humidity range of 32–90% RH, respectively. The vibration is asserted to the device through a hole on the bottom of the environmental chamber, which creates inhomogeneity of temperature distribution within the chamber. Sivakumar et al. characterized the accrual rate of stiction and mechanical fatigue in a MEMS micromirror device within a temperature and humidity range of 25 °C to 90 °C and 20% to 80% RH, respectively [5]. Lin et al. studied the RF MEMS switches performance and reliability under different temperatures [6]. Under temperature aging, the calculated Mean Time to Failure (MTTF) for each condition is 985 h, 822 h and 751 h for the temperatures of 50 °C, 85 °C and 105 °C, respectively.

Despite the great need of studying the MEMS reliability under different environmental conditions, one of the major challenges is to conduct those tests with different environmental factors using standard MEMS testing equipment, such as interferometer, laser-Doppler vibrometer, vibration table (piezo shaker), and different microscopes, etc. Some of the MEMS testing equipment is too bulky to be operated in the commercially available environmental chambers or ovens, while some other equipment is highly sensitive to environmental factors such as the piezo shakers. Taking piezo shaker as an example, the performances of piezoelectric materials are strongly influenced by temperature, especially around the Curie temperature of the material [7]. Therefore, it is difficult to use the piezo shaker within an oven. Meanwhile, most of the piezo shakers can generate very limited force, often in the range of tens of Newton, thus it is equivalently difficult to mount an oven, even the lightest one, on the piezo shaker. Such dilemma might be the detrimental factor for the lack of reliability of vibration energy harvesters (VEHs), in spite of a great deal of effort in developing various novel VEH devices [8,9,10,11].

Therefore, there is a strong need and market pull for standard generators of different environmental condition (environmental chambers), which can be integrated on different standard MEMS test equipment. For this purpose, the environmental chamber must be compact, lightweight and capable of generating a wide range of environmental conditions. Towards these goals, the major technical challenge is to integrate all functional elements within a rather small volume and not to exceed the mass and volume limitation. In addition, with miniaturized heating and humidifying elements, it is not easy to realize heating and humidifying functions with reasonable range and speed, compared to their macroscopic counterparts. Although control algorithms can be utilized to optimize the heating and humidifying process, most of the reported algorithms are developed for standard “big” environmental chambers [12,13,14]. Their applicability on miniaturized systems still need to be investigated.

In this paper, we proposed a miniaturized reliability test system for microdevices with controlled environmental parameters, which has the advantages of lightweight, small volume, wide and stable range of controllable environmental parameters. The system can be integrated with the piezo shaker, white light interferometer and other equipment to complete the performance characterization of microdevices under different environmental conditions. The technical merits of the proposed system are to integrate all functional components within a very limited space, and fulfill all heating, humidifying and measurement functionalities comparable to bulky environmental systems. Although the test system in this paper is a specific system, it provides a technical platform for the reliability test of microdevices and can be widely used.

## 2. System Design

### 2.1. Overal System Design

The concept of the proposed test system is to control the environmental parameters around the Device Under Test (DUT) while other performance of the DUT is tested. This requires the test chamber to be highly miniaturized and lightweight, in order to be integrated with other characterization tools for microdevices. It is especially crucial for the reliability characterization of Vibration Energy Harvesters (VEHs), because the test chamber needs to be mounted on the piezo shaker, whose loading capability is strongly limited in terms of pushing force. Objects with large mass will deteriorate the performance of the shaker significantly.

**Figure 1:**
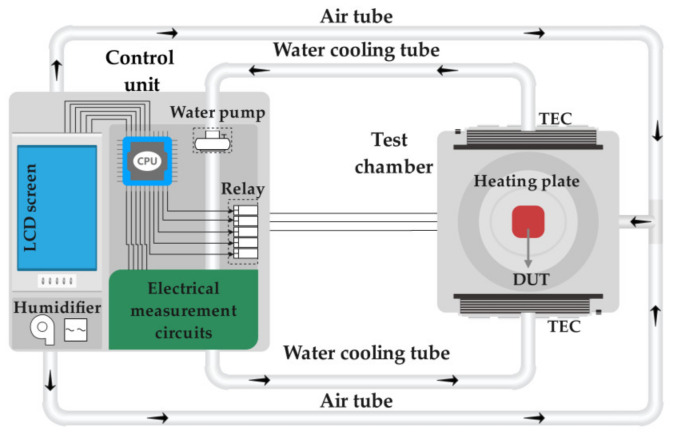
Schematic illustration of the miniaturized reliability test system with controlled environment parameters.

The proposed reliability test system is a miniaturized and lightweight equipment, based on a structure illustrated schematically in Figure 1. The system is composed of two main components: a test chamber and a control unit, which are connected by electrical cables and tubes. The test chamber is a miniaturized metallic chamber capable of changing its internal environmental parameters, mainly temperature and humidity, accurately and rapidly. The test chamber is highly compact and lightweight, equipped with electrical feedthrough and optical observation window, enabling its integration to standard characterization equipment such as white light interferometer, laser Doppler vibrometer and piezo shaker. The control unit includes a humidity regulator, temperature and humidity control circuits, electrical measurement circuits and a human–computer interaction module. The electrical measurement circuits include mainly components for capacitive and resistive measurements, and will not be detailed in this paper.

More specifically, the temperature regulator consists of a ceramic heating plate with a power of 96 W and two heat-resistant thermoelectric coolers (TECs) based on Peltier effect with power of 36 W. To achieve a better cooling while maintaining a designated volume of the test chamber, water cooling is used to dissipate heat from the hot end of the TEC. The humidity in the test chamber is controlled by using an ultrasonic transducer. The fan transmits the water vapor with a certain controlled humidity to the test chamber through the plastic tube. The interaction module consists of an LCD display screen and a STM32 microprocessor. The LCD screen displays the temperature and humidity data in the test chamber as well as the capacitance and resistance measured by the measurement module. By touching the LCD screen, control commands are sent to the temperature and humidity control module, which controls the ceramic heating plate, TECs and humidifier to change the temperature and humidity in the test chamber.

### 2.2. Test Chamber Design

**Figure 2:**
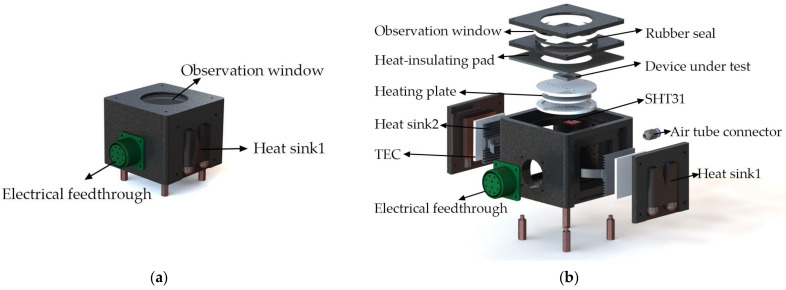
(**a**) Schematic illustration of the assembled test chamber, (**b**) schematic illustration of the exploded structure of the test chamber.

In order to achieve maximum heating and humidifying capacity with minimum size and weight, the test chamber must be carefully designed, and elements and their layout within the chamber must be optimized. A schematic illustration of the designed test chamber is shown in Figure 2a, and an exploded view of the test chamber is detailed in Figure 2b. In order to maximize the efficiency of the system, the temperature regulator is directly installed within the test chamber. The ceramic heating plate is sandwiched between two sample stages, and the TECs are installed on both sides of the test chamber. A temperature and humidity sensor (Sensirion SHT31) is installed on the bottom side of the sample stage to monitor the real-time temperature and humidity in the closed vicinity of DUT. During measurements, the DUT is mounted on the top surface of the sample stage, with several mounting and pin-configuration possibilities enabled by pre-defined mounting plugs. For the heating processes, the sample stage will quickly transfer heat between the ceramic heating plate and the DUT. For the cooling processes, the hot end and the cold end of TEC will release heat and absorb heat respectively, due to the Peltier effect. Heat sink 2 mounted on the cold end of the TECs is used to quickly transfer heat between the cold end of the TEC and the chamber, while heat sink 1 mounted on the hot side of the TEC removes heat from the hot end of the TEC through water cooling. The combination of the passive and active water-cooled heatsinks enabled maximum efficiency and stability for the cooling processes. The mounting sites of the TECs and heatsinks are chosen under the criteria of shortest distance to the DUT. For the humidity control processes, water vapor with a controlled humidity enters the test chamber through the tube connector at the back of the chamber. The humidity in the water vapor is adjusted by using an ultrasonic transducer installed within a water tank inside the control unit. The entire model design is based on a symmetrical rule to ensure the homogeneity of temperature and humidity within the test chamber.

### 2.3. Control Unit Design

**Figure 3:**
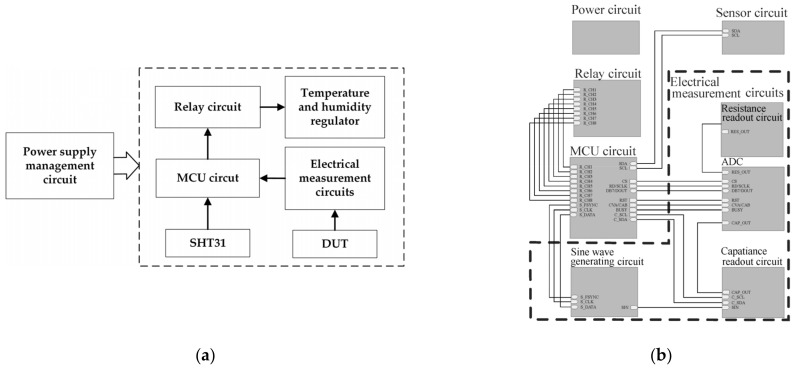
(**a**) Block diagram of the control circuit, (**b**) schematic illustration of the control circuit.

The control unit of the test system includes a power management circuit, an MCU circuit, a relay circuit, a sensing circuit and the electrical measurement circuits. Due to the complexity of the entire circuit system, a hierarchical design approach is adopted to construct the system from modular components with logic orders. A schematic illustration of the overall circuit is shown in Figure 3. The power management circuit provides power with different voltage levels to the various components in the system with different power requirements. The MCU circuit is used to realize all the designated functions of the STM32 microprocessor, including executing, uploading and downloading programs, implementing all the calculation and control algorithms, as well as communicating with other components. The relay circuit isolates the low-voltage side from the high-voltage side of the circuits by using an optocoupler, and controls the operation of the temperature and humidity regulator by using a high-power MOSFET as a switch. The sensor circuit drives the SHT31 temperature and humidity sensor to measure the real-time temperature and humidity data within the test chamber. The electrical measurement circuits measure the capacitance, resistance and other physical parameters of the DUT. In the circuit layout, the circuits are partitioned according to different voltage levels and functions. At the same time, single-point grounding of both the analog ground and digital ground is implemented to isolate the analog circuit from the digital circuit, avoiding the crosstalk between them. These design and structural concepts guaranteed the accuracy and reliability of the proposed test system.

### 2.4. Control Algorithms

**Figure 4:**
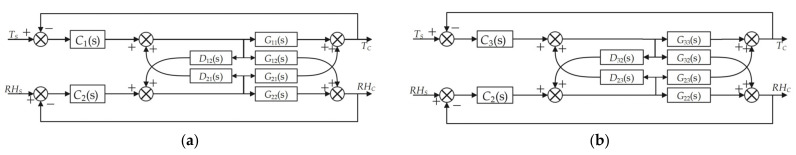
(**a**) The heating-humidifying decoupling control block diagram, (**b**) the cooling-humidifying decoupling control block diagram.

Rapid control of temperature and humidity simultaneously under limitations of mass and volume is challenging, due to limited choice of components, strong temperature/humidity coupling, performance hysteresis and nonlinear dynamic characteristics. Consequently, a simple PID controller will not be sufficient to control the system [14,15]. Therefore, the proposed test system utilizes a feed-forward decoupling control algorithm to decouple and control the temperature and humidity, improving the accuracy of the system [16]. The feed-forward decoupling control algorithm is used to predict the effect of the coupling between the temperature and humidity on the control process, and subsequently eliminate the cross-coupling effect. The block diagram of the heating-humidifying decoupling controller is shown in Figure 4a. Similarly, the cooling-humidifying decoupling control block diagram is obtained, as shown in Figure 4b.

It is crucial for the feed-forward decoupling control system to solve the feed-forward decoupling transfer function. The output of the heating-humidifying decoupling system can be described as:

(1) $$\left[T_{C}\right.\left.RH_{C}\right]=\left[\begin{array}{cc} C_{1}\left(s\right) & C_{2}\left(s\right) \end{array}\right]\times \left[\begin{array}{cc} G_{11}\left(s\right) & G_{12}\left(s\right)+G_{22}\left(s\right)\times D_{12}\left(s\right)\\ G_{21}\left(s\right)+G_{11}\left(s\right)\times D_{21}\left(s\right) & G_{22}\left(s\right) \end{array}\right]$$

where *G*_11_(s) is the system heating model and *G*_12_(s) is the system heating-humidifying coupling model. *G*_21_(s) is the system humidifying-heating coupling model, *G*_22_(s) is the system humidifying model, *D*_21_(s) is the humidifying-heating feed-forward decoupling factor and *D*_12_(s) is the heating-humidifying feed-forward decoupling factor.

After the decoupling the heating and humidifying process, the ideal system output should be described as:

(2) $$\left[T_{C}\right.\left.RH_{C}\right]=\left[\begin{array}{cc} C_{1}\left(s\right) & C_{2}\left(s\right) \end{array}\right]\times \left[\begin{array}{cc} G_{11}\left(s\right) & 0\\ 0 & G_{22}\left(s\right) \end{array}\right]$$

From Equations (1) and (2), the relationship between the heating, humidifying feed-forward decoupling factor and the inherent model of the system can be obtained:

(3) $$\left[\begin{array}{cc} D_{21}\left(s\right) & 1\\ 1 & D_{12}\left(s\right) \end{array}\right]\times \left[\begin{array}{cc} G_{11}\left(s\right) & G_{12}\left(s\right)\\ G_{21}\left(s\right) & G_{22}\left(s\right) \end{array}\right]=\left[\begin{array}{cc} 0 & 0\\ 0 & 0 \end{array}\right]$$

Similarly, the expression between the feed-forward decoupling factor of cooling, humidifying and the inherent model of the system can be obtained:

(4) $$\left[\begin{array}{cc} D_{32}\left(s\right) & 1\\ 1 & D_{23}\left(s\right) \end{array}\right]\times \left[\begin{array}{cc} G_{22}\left(s\right) & G_{23}\left(s\right)\\ G_{32}\left(s\right) & G_{33}\left(s\right) \end{array}\right]=\left[\begin{array}{cc} 0 & 0\\ 0 & 0 \end{array}\right]$$

where, *G*_23_(*s*) is humidifying-cooling coupling model, *G*_32_(s) is cooling-humidifying coupling model, *G*_33_(s) is the system heating model, *D*_23_(s) is humidifying-cooling feed-forward decoupling factor and *D*_32_(s) is cooling-humidifying feed-forward decoupling factor.

### 2.5. Simulink Simulation

**Figure 5:**
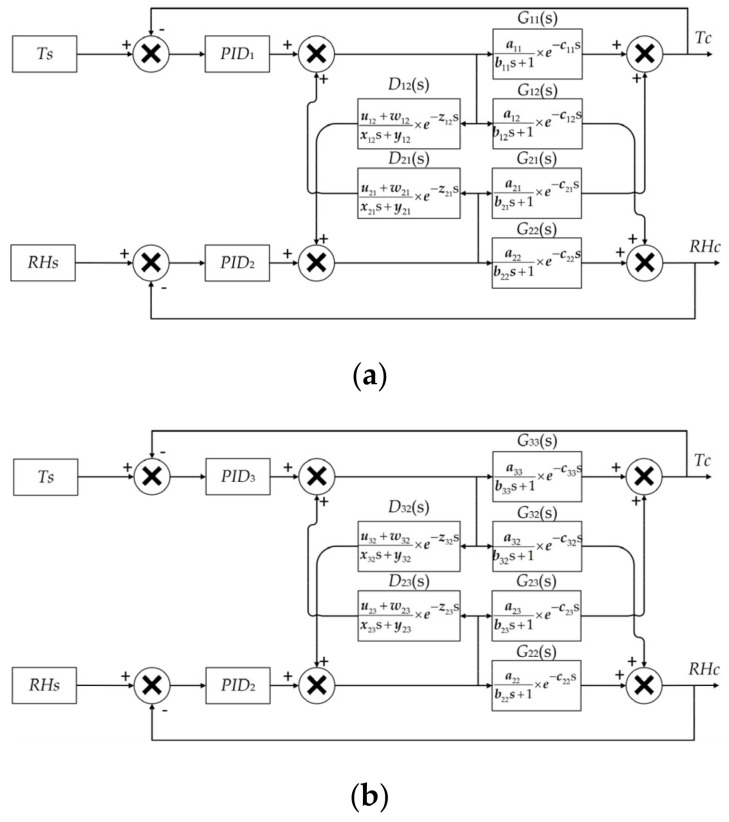
(**a**) The heating-humidifying system model, (**b**) the cooling-humidifying system model [16].

To verify the effectiveness of the control methodology, the control system is simulated by using Simulink according to the proposed control block diagram in Figure 5.

The model of the control system is effective in tuning the parameters of the controller, including both the feedback and feed-forward parameters, which are essential factors for a rapid and accurate control of temperature and humidity. In the actual tuning process, the parameters *a*, *b*, *c* of *G*_11_(s), *G*_12_(s), *G*_21_(s), *G*_23_(s) and *G*_33_(s) can be obtained experimentally [17], and the parameters *u*, *w*, *x*, *y*, *z* of feed-forward decoupling factors *D*_21_(s), *D*_12_(s), *D*_23_(s) and *D*_32_(s) can be solved by Equations (3) and (4). The solving process will be used for the tuning process detailed in Section 3.2. By implementing the discretized feed-forward decoupling factor differential equation into the control program, the effects of the system caused by the temperature and humidity coupling can be predicted and eliminated [18].

## 3. Results and Discussion

### 3.1. Manufacturing Results

**Figure 6:**
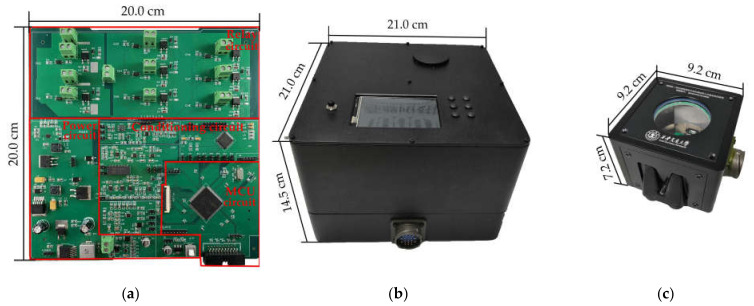
Photos of (**a**) the circuit board, (**b**) the control chamber, (**c**) the test chamber.

The photos of the manufactured circuit board, control unit and test chamber are shown in Figure 6. The temperature and humidity controllers are connected to the KF-2.54 terminal in the relay circuit. To drive multiple high-power devices at the same time, the PCBs in the system involve design with windowed topology and heat dissipation holes, in order to increase the heat dissipation capacity of the circuit board. The control unit is fabricated by 3D printing technology, and the upper cover is equipped with switch buttons, control buttons, an interaction interface, and a water inlet for the humidity regulator. An optical observation window is installed on the top surface of the test chamber to enable the optical measurements on the DUT. The housing of the test chamber is made of aluminum alloy with surface treatment of thick anodization, in order to enhance corrosion resistance. The test chamber has external dimensions of 9.2 cm × 9.2 cm × 7.2 cm and a weight of 1.07 kg. The control unit and the test chamber are connected through high-temperature aviation plugs for reliable electrical connections.

### 3.2. System Tuning

**Figure 7:**
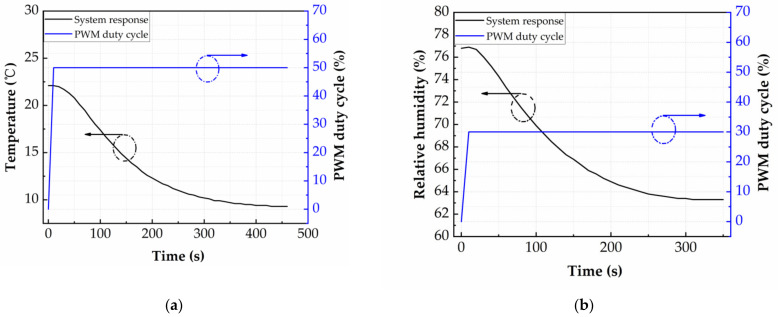
(**a**) Temperature response of the test system to a 50% PWM duty-cycle step cooling signal. (**b**) Relative humidity response of the test system to a 30% PWM duty-cycle step cooling signal.

In order to solve the temperature and humidity model of the test chamber, the temperature and humidity open-loop response and the temperature and humidity open-loop coupling response of the test chamber are studied in time domain [19]. Figure 7a, b show the temperature open-loop response of the test system to a 50% PWM duty-cycle step cooling signal and humidity open-loop coupling response of the test system to a 30% PWM duty-cycle step cooling signal, respectively (PWM cycle is 1 s). The 50% PWM duty-cycle step cooling signal is given at 0 s with a rise time of 20 ms. The temperature change of the test chamber delay is 44 s (warming up of the cooler), then rapidly from 44 s to 250 s (cooling process), and finally gradually between 250 s and 450 s. At the initial temperature of 20 °C, the maximum cooling rate is 0.075 °C per second at 80 s, and the temperature of the test chamber is finally stabilized at 9.3 °C under a 50% PWM duty-cycle step cooling signal. The open-loop coupling response of the humidity within the chamber follows a similar pattern. The 30% PWM duty-cycle step cooling signal is given at 0 s with a rise time of 20 ms. The humidity change of the test chamber delay is 28 s, then rapidly from 28 s to 200 s, and finally gradually between 200 s and 350 s. At the initial humidity of 76.8% RH, the maximum dehumidifying rate is 0.095% RH per second at 50 s, and the humidity of the test chamber is finally stabilized at 63.3% RH under a 30% PWM duty-cycle step cooling signal. The coupling of cooling and humidity is mainly caused by the sudden drop of temperature at the TEC cold end, which results in condensation of water vapor in the air. Similarly, we can get the open-loop response curve and open-loop coupling response curve of heating and humidifying, which will not be repeated here.

The two-point method was used to obtain the parameters of first-order delay models of the system, as given in Table 1.

Through Equations (3) and (4), the parameters of the transfer functions of feed-forward decoupling factor are derived, as shown in Table 2.

These parameters of transfer functions of the proposed system can be used to model the response of the control system. The analytical form of the model enabled the modeling process to be simple and accurate, where key design parameters can be tuned readily and conveniently.

**Figure 8:**
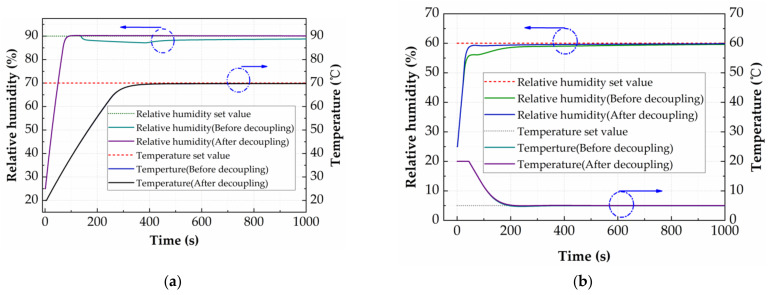
(**a**) Response curve of heating-humidifying system before and after decoupling. (**b**) Response curve of cooling-humidifying system before and after decoupling.

The PID Tuner is utilized to solve the PID parameters in the system. The comparison between the system responses before and after the introduction of the feed-forward decoupling factor is shown in Figure 8. The target temperature of 70 °C is realized within the time of 400 s in Figure 8a and the target temperature of 5 °C is realized within the time of 200 s in Figure 8b, both showing no overshoot or oscillation behavior. The temperature response curves before and after decoupling in Figure 8a, b almost coincide, which means the feed-forward decoupling factor has almost no effect on the temperature regulation of the system. However, the feed-forward decoupling factor can obviously improve the humidity control of the system. In the heating-humidifying system of Figure 8a, the target humidity of 90% RH is realized within the time of 100 s, showing no overshoot or oscillation behavior. However, when the system temperature reaches 45.6 °C at 146 s, the humidity of the system before decoupling decreases at the rate of 0.004% RH per second until 420 s, and then increases to 88.7% RH at 1000 s. Compared with before decoupling, the humidity of the system after decoupling is always stable at a target humidity of 90% RH. In the cooling-humidifying system in Figure 8b, by introduction of the feed-forward decoupling factor, the regulating time decreases from 323 s to 108 s. To sum up, the simulation results show that the feed-forward decoupling factor has almost no effect on the temperature regulation of the system, but can obviously improve the humidity regulation.

### 3.3. System Characterization

From the simulation in the previous section, it can be seen that humidification has little effect on refrigeration and heating, so only feed-forward decoupling factors *D*_12_(s) and *D*_32_(s) are introduced in the system. In order to introduce the feed-forward decoupling factor into the control system, we need to carry out Z transformation on the feed-forward decoupling factors, and then they are transformed into difference equations, finally.

**Figure 9:**
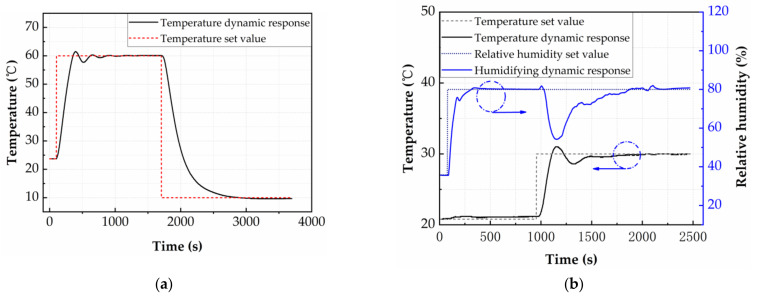
(**a**) System temperature dynamic response. (**b**) System temperature-humidity coupling dynamic response.

By introducing the difference equations into the control program, the feed-forward decoupling factor of the actual control system is added. The PID parameters obtained from the simulation are brought into the actual system, and then fine-tuning is carried out to obtain the temperature control effect of the system shown in Figure 9a and the temperature and humidity decoupling control effect shown in Figure 9b.

In order to characterize the heating dynamic adjustment performance of the system, a step temperature signal that has a target temperature of 60 °C is given at 100 s, as shown in Figure 9a. The target temperature of 60 °C is realized within the time of 2023 s and the maximum overshoot is 2.5%. The system temperature differs from the target temperature by 0.1 °C within 700 s, and gradually stabilizes at 60 °C. The characterization of the cooling dynamic adjustment performance of the system within the chamber follows a similar pattern. A step temperature signal that has a target temperature of 10 °C is given at 1706 s, as shown in Figure 9a. The temperature of the test chamber decreases rapidly from 1706 s to 2300 s (cooling process), and finally gradually between 2300 s and 2700 s (precise temperature control to avoid overshoot). The target temperature of 10 °C is realized within the time of 1080 s, showing no overshoot or oscillation behavior. The system temperature differs from the target temperature by 0.1 °C within 1124 s. The maximum temperature change rate is 0.2 °C/s for the heating process and −0.175 °C/s for the cooling process. Compared to the simulation, the actual temperature dynamic response of the system is slower because of the simplified first-order heating and cooling model, but can meet the requirements of most test devices.

The anti-disturbance test of the system is shown in Figure 9b, and a step humidity signal that has a target humidity of 80% RH is given at 176 s. The target humidity of 80% RH is realized within the time of 234 s, and the maximum overshoot is 1%. The system humidity differs from the target humidity by 0.2% RH within 474 s. During the humidification process, the system temperature first gradually rises in 40–308 s, and stabilizes at 21.2 °C in 380–410 s, and then gradually returns to the initial value. The reason for this phenomenon is the temperature of the atomizing gas will be slightly higher than the temperature in the test chamber. A step temperature signal that has a target temperature of 30 °C is given at 1054 s. Due to the feedforward decoupling factor, the system humidity will increase in advance before being affected by temperature. Therefore, the system humidity will be higher than the set value of 80% RH in 1074–1126 s, and then as the temperature continues to rise to 31 °C, the system humidity gradually decreases to 54.3% RH. As the system temperature gradually stabilizes, the system humidity is also slowly increasing, and finally stabilizes at 80% ± 1% RH at 2182 s. Compared to the simulation, during the system temperature increase, the internal temperature of the test chamber does not reach complete consistency, which causes the system humidity to fail to maintain the set value.

**Figure 10:**
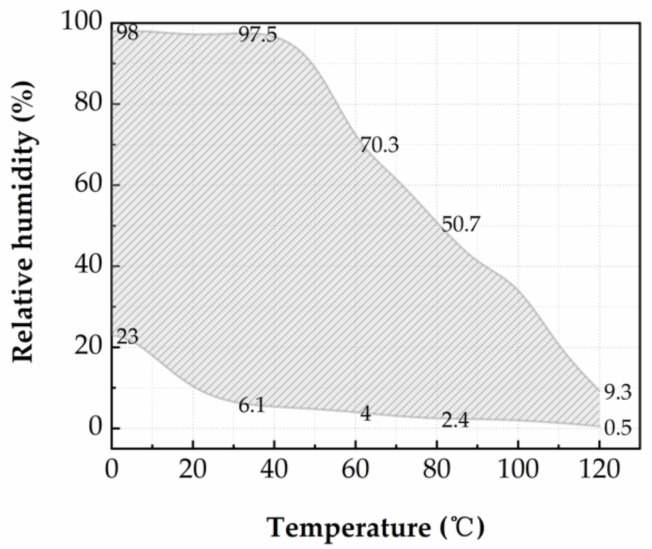
Relative humidity tuning characteristics in the test chamber as a function of temperature.

In order to study the static tuning range of the system, the humidity is set to 100% RH and the temperature is increased from 0 °C to 120 °C, with a step of 10 °C. When the system is in steady state, the maximum humidity of the system is recorded. As a next step, the humidity is set to 0% RH and the temperature is decreased from 120 °C to 0 °C, with a step of 10 °C. When the system is in steady state, the minimum humidity of the system is recorded. The measured static tuning range of the system is shown in Figure 10. In the temperature range of 0–40 °C, the maximum humidity that can be reached in the test chamber is 97% RH. The maximum humidity tuning range of 4.8–97% RH in the chamber is realized at a temperature of 40 °C. At elevated temperature, the maximum humidity within the chamber decreases. The measured maximum humidity is 70.3% RH at 60 °C, 40% RH at 90 °C and 9.3% RH at 120 °C.

The maximum humidity in the chamber decreases rapidly when the temperature increases, which can be attributed to the following reasons: As the temperature increases, the saturated vapor is capable of holding more liquid content. The maximum level of liquid content in the vapor generated by the humidifier (held at room temperature) cannot exceed the level of liquid content in the saturated vapor. The minimum humidity of the system is 0.5% RH at 120 °C and 23% RH at 0 °C, which because of the moisture content in saturated air will decrease as the temperature decreases. Considering the insufficiencies above, there is still space for improvements in the humidification system. For example, different humidification devices can be used at different temperature ranges, i.e., ultrasonic atomization device can be used to humidify the chamber at a low-temperature range, and thermal vaporization humidifier at a high-temperature range.

### 3.4. System Functionality Verification

**Figure 11:**
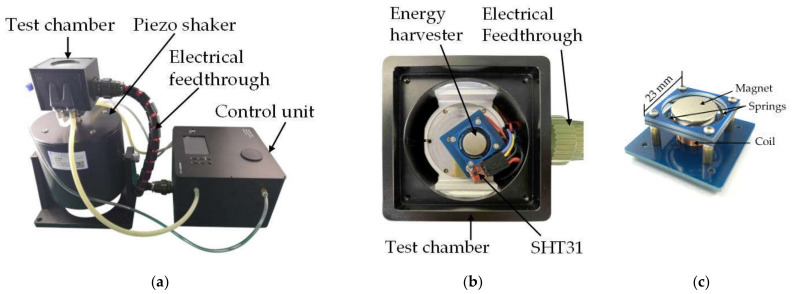
Photos of (**a**) the integration of the miniaturized reliability test system with controlled environment parameters and the piezo shaker, (**b**) the energy harvester in the test chamber and (**c**) the energy harvester device.

Figure 11a shows a photo of the miniaturized reliability test system integrated with a piezo shaker. Such experimental configuration enables the reliability test of micro-energy harvesters, which have not been reported due to the incompatibility between the piezo shaker and environmental chambers (e.g., a convection oven). The shaker used in this study is a model JZK-20 piezo shaker from Sinocera Corporation, which is capable of generating a maximum excitation force of 200 N. The DUT is mounted on a customized sample stage with electrical connections to the feedthrough on the sidewall of the chamber, as shown in Figure 11b. The sample stage can be readily exchanged to adapt to different types of device connections. A second temperature sensor is mounted above the sample stage next to the DUT in order to monitor the actual temperature around the DUT. The DUT is a customized electromagnetic vibration energy harvester fabricated by the authors for the verification of the proposed test system. It is based on a flex-rigid structure, in which an NdFeB disc magnet is suspended over a winded coil, by four polyimide springs. The key geometric parameters of the device are shown in Table 3. The working principle of the energy harvester is to convert the movement between the coil and the magnet into an induced voltage in the coil. When the resonant frequency of the energy harvester is matched with the external vibration frequency, the output voltage of the energy harvester reaches the maximum. Due to the complex rigid-flex structure and the polymer nature of the springs, it is very interesting to study the reliability of the energy harvester in different environmental conditions. Therefore, the energy harvester is selected as a typical scenario to verify the functionality of the system. As a proof-of-concept experiment, only simple behavioral tests of the energy harvester at different temperature and humidity were conducted.

**Figure 12:**
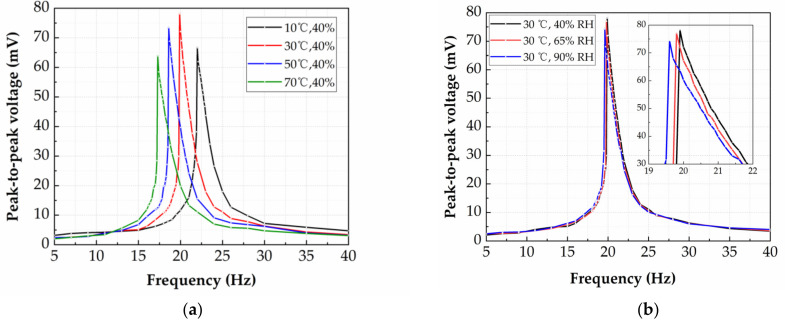
(**a**) The influence of temperature on the output voltage and resonant frequency of the energy harvester. (**b**) The influence of humidity on the output voltage and resonant frequency of the energy harvester.

Firstly, the effect of temperature on the dynamic response of the energy harvester is studied. The humidity inside the chamber is set to be 40% RH, and the temperature is set to 10 °C, 30 °C, 50 °C and 70 °C, respectively. The output voltage is measured by exciting the energy harvester with a sinusoidal acceleration of 0.3 g (±0.15 g). The influence of temperature on the output voltage and resonant frequency of the energy harvester is shown in Figure 12a. The horizontal axis represents the vibration frequency of the piezo shaker, and the vertical axis represents the peak-to-peak voltage of the energy harvester. It can be seen that when the temperature increases, the resonance frequency of the energy harvester shifts towards the low-frequency regime, as a result of the reduced spring stiffness of the polyimide at increased temperature values. The humidity in the test chamber is held constant at 40% RH throughout the experiments. For the temperature of 10 °C, the maximum output voltage of the energy harvester is 66.6 mV at 22.0 Hz. For the temperature of 30 °C, the maximum output voltage of the energy harvester is 78.1 mV at 19.9 Hz. For the temperature of 50 °C, the maximum output voltage of the energy harvester is 73.4 mV at 18.6 Hz. For the temperature of 70 °C, the maximum output voltage of the energy harvester is 63.9 mV at 17.3 Hz. In theory, the output voltage of the energy harvester should increase once the spring stiffness decreases. However, an increase of the output voltage is firstly observed from 10 °C to 30 °C, followed by a decrease of the output voltage firstly observed from 30 °C to 70 °C. This behavior is repeatedly observed in the experiments, but has not been sufficiently explained yet. More thorough study will be conducted to understand this phenomenon in the future.

Secondly, the effect of humidity on the dynamic response of the energy harvester is studied. The temperature in the chamber is set to 30 °C, and the humidity is set to 40%, 65% and 90% RH, respectively. The output voltage is measured by exciting the energy harvester with a sinusoidal acceleration of 0.3 g (±0.15 g). The influence of humidity on the output voltage and resonance frequency of the energy harvester is measured, after maintaining the temperature and humidity at constant value for one hour, as shown in Figure 12b. For the temperature of 30 °C, the resonance frequency of the harvester is decreased by 0.3 Hz in the humidity range of 40–90% RH. This is induced by the slight mass enhancement of the magnet due to the condensation of water vapor in a high relative humidity environment. The experimental results show that at a temperature of 30 °C, humidity has little effect on the output performance of the energy harvester in a short period of time. This is due to the excellent water-resistant properties of the polyimide springs. Under high-temperature and long-term conditions, the effect of different humidity on the output performance of the energy harvester needs to be further investigated.

## 4. Conclusions

In this paper, a miniaturized reliability test system with controlled environment parameters has been designed, simulated, manufactured and characterized. The system can be integrated with the piezo shaker and other equipment under the condition of controllable temperature and humidity to characterize the test devices in real time. The first-order model of the system was established and verified in Simulink. The presented test system is capable of generating a temperature range of 0–120 °C and a humidity range of 20–90% RH (0–55 °C), and the control effect of temperature static error and humidity static error were ±0.1 °C and ±1% RH (30 °C), respectively. The functionality of the proposed test system was verified by integrating with a piezo shaker to test the environmental reliability of an electromagnetic vibration energy harvester. The system is the first-generation product with controllable environmental parameters, and further research will be carried out on high-temperature humidification and frosting caused by rapid cooling of the system.

## Figures and Tables

**Table 1:** Parameters of first-order delay models of the system.

Parameters	Values	Parameters	Values	Parameters	Values
*a*_11_	0.1559	*b*_11_	751.27	*c*_11_	5
*a*_12_	0.3357	*b*_12_	1618.00	*c*_12_	136
*a*_21_	0.0004	*b*_21_	428.45	*c*_21_	100
*a*_22_	0.1949	*b*_22_	183.65	*c*_22_	13
*a*_23_	0.0050	*b*_23_	976.46	*c*_23_	105
*a*_32_	0.0488	*b*_32_	107.60	*c*_32_	28
*a*_33_	0.0270	*b*_33_	124.80	*c*_33_	44

**Table 2:** Parameters of the transfer functions of feed-forward decoupling factor.

Parameters	Values	Parameters	Values	Parameters	Values	Parameters	Values	Parameters	Values
*u*_12_	−61.6500	*w*_12_	−0.3357	*x*_12_	315.30	*y*_12_	0.1949	*z*_12_	135
*u*_21_	−0.7036	*w*_21_	−0.0009	*x*_21_	1599.00	*y*_21_	0.3639	*z*_21_	95
*u*_23_	0.6205	*w*_23_	−0.0050	*x*_23_	26.36	*y*_23_	0.0270	*z*_23_	65
*u*_32_	−8.9690	*w*_32_	−0.0488	*x*_32_	20.97	*y*_32_	0.1949	*z*_32_	135

**Table 3:** Parameters of the electromagnetic vibration energy harvester.

Parameters	Measured Values
Thickness of the magnet (mm)	3.0
Diameter of the magnet (mm)	15.0
Thickness of the spring (mm)	0.2
Length of the spring (mm)	12.6
Width of the spring (mm)	1.0
Coil–magnet distance (mm)	6.4
Number of coil turns	287

## References

[1] Hartzell A.L., Da Silva M.G., Shea H.R., Senturia S.D., Howe R.T., Ricco A.J. (2010). In-Use Failures. MEMS Reliability.

[2] Pustan M., Birleanu C., Serdean F. (2017). Impact of environmental conditions on the reliability of MEMS components from optical applications. MATEC Web Conf..

[3] Zhang Z., Liao X. Sensitivity characteristics in the packaged inline RF MEMS power sensors under different temperature and humidity environments. Proceedings of the 2012 IEEE Sensors.

[4] Jan M.T., Ahmad F., Hamid N.H.B., Khir M.H.B.M., Shoaib M., Ashraf K. (2016). Experimental investigation of temperature and relative humidity effects on resonance frequency and quality factor of CMOS-MEMS paddle resonator. Microelectron. Reliab..

[5] Sivakumar G., Ranganathan R., Gale R., Dallas T. Reliability study of a MEMS array under varying temperature and humidity conditions. Proceedings of the Reliability, Packaging, Testing and Characterization of MEMS/MOEMS and Nanodevices IX.

[6] Lin T., Paul S., Lu S., Lu H. (2009). A study on the performance and reliability of magnetostatic actuated RF MEMS switches. Microelectron. Reliab..

[7] Wu J., Gao X., Chen J., Wang C., Zhang S., Dong S. (2018). Review of high temperature piezoelectric materials, devices, and applications. Acta Physica Sinica.

[8] Wei C., Jing X. (2017). A comprehensive review on vibration energy harvesting: Modelling and realization. Renew. Sustain. Energy Rev..

[9] Wang J., Zhou S., Zhang Z., Yurchenko D. (2019). High-performance piezoelectric wind energy harvester with Y-shaped attachments. Energ. Convers Manag..

[10] Toshiyoshi H., Ju S., Honma H., Ji C.H., Fujita H. (2019). MEMS vibrational energy harvesters. Sci. Technol. Adv. Mater..

[11] Tan Y., Dong Y., Wang X. (2017). Review of MEMS Electromagnetic Vibration Energy Harvester. J. Microelectromech. S..

[12] Hung S., Lin A.C., Chen Y. Developing the intelligent predetermined control method for a new temperature and humidity chamber: A Case study for rising temperature under constant relative humidity. Proceedings of the IEEE International Conference on Mechatronics and Automation (ICMA).

[13] Hu M.Y., Fang K.L. (2013). The Temperature and Humidity Control of Artificial Climate Chamber Based on Feed-Forward Compensation Decoupling. Adv. Mater. Res..

[14] Li M., Wei J., Shen T. Temperature and humidity decoupling control for enthalpy difference Laboratory. Proceedings of the Chinese Automation Congress (CAC).

[15] Long W., Li F., Luo L., Zhang X. The design of temperature and humidity Control System for Incubation based on data fusion and fuzzy decoupling. Proceedings of the IEEE International Conference on Mechatronics and Automation (ICMA).

[16] Wang L., Zhu Z. (2020). Research on Temperature and Humidity Decoupling Control of Constant Temperature and Humidity Test Chamber. IOP Conf. Ser. Mater. Sci. Eng..

[17] Quoilin S., Aumann R., Grill A., Schuster A., Lemort V., Spliethoff H. (2011). Dynamic modeling and optimal control strategy of waste heat recovery Organic Rankine Cycles. Appl. Energ..

[18] Chen G.N., Li X., Fang K.L. (2013). The Humidity and Temperature Control of Immersion Cycle Corrosion Test Chamber. Adv. Mater. Res..

[19] Li J., Dongmei Y., Zhou R., Zhang H. A humidity control system based on T&H-decoupling and PID self-tuning fuzzy algorithm. Proceedings of the IEEE International Conference on Electronic Measurement & Instruments (ICEMI).

